# Brugada Syndrome Manifested by the Typical Electrocardiographic Pattern both in the Right Precordial and the High Lateral Leads

**Published:** 2008-04-01

**Authors:** Hamid Bonakdar, Majid Haghjoo, Mohammad Ali Sadr-Ameli

**Affiliations:** Department of Pacemaker and Electrophysiology, Rajaie Cardiovascular Medical and Research Center, Iran University of Medical Sciences, Tehran, Iran

**Keywords:** Brugada syndrome, electrocardiography, ST-segment elevation, lateral leads

## Abstract

We identified a patient with the Brugada syndrome and frequent episodes of the traumatic syncope. This patient presented with alternating ST-segment elevation in the right precordial and the high lateral leads. The signal-averaged ECG was positive for the late potentials and electrophysiology study revealed no inducible supraventricular or ventricular tachycardias. Because of the frequent traumatic syncope, a dual-chamber implantable cardioverter-defibrillator was implanted. This report suggests that the Brugada syndrome may have different electrocardiographic presentations within a single individual over a short period of time. The significance of these changes needs to be assessed in a prospective long term study.

## Introduction

The Brugada syndrome is a distinct type of the idiopathic ventricular fibrillation, which characterized by ST-segment elevation in right precordial leads (V1-V3), right bundle branch block (RBBB) pattern, and high incidence of sudden cardiac death (SCD) in patients with structurally normal hearts [1]. Recently it was reported that typical electrocardiographic pattern of the Brugada syndrome may be observed intermittently in the right precordial and the inferior leads [2-7].

In present report, we describe the clinical, electrocardiographic and electrophysiologic characteristics of a patient with Brugada syndrome who presented with alternating typical coved type ST-segment elevation in right precordial leads and lateral leads.

## Case Report

**Figure 1:**
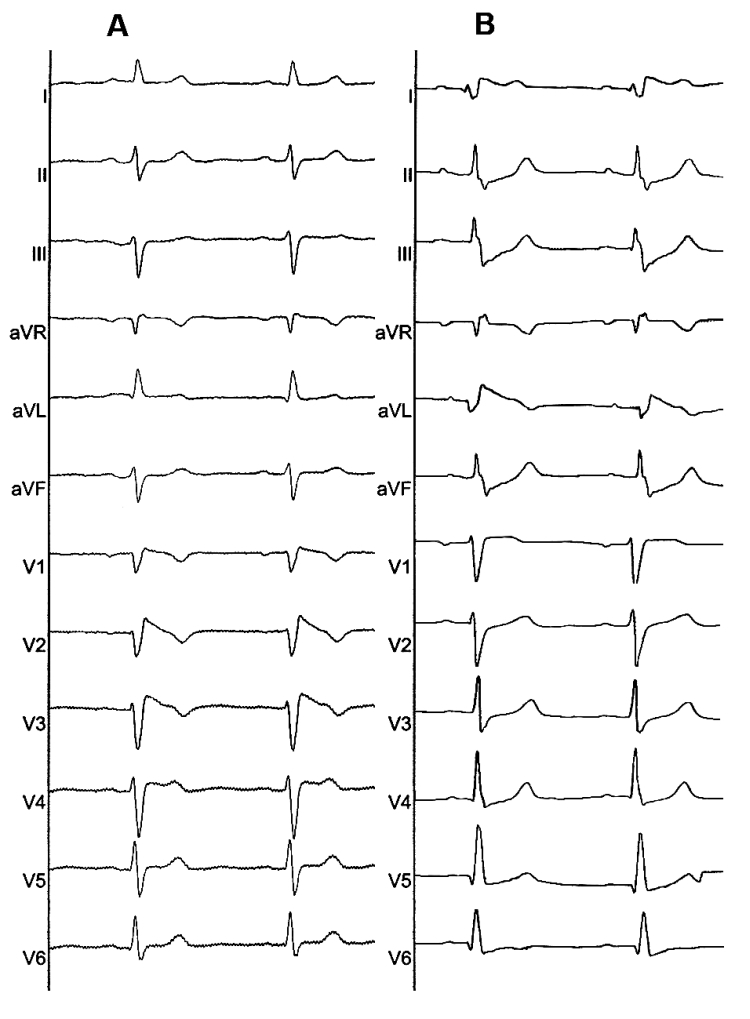
**A,** twelve lead ECG obtained on admission showed typical coved-type ST-segment elevation in the right precordial leads (V1-V3). **B,**  twelve lead ECG obtained two days later revealed ST-segment elevation in the high lateral leads (I, aVL)

A 60-year-old female, with no structural heart disease, was referred to our center for the evaluation of frequent episodes of traumatic syncope. The patient had diabetes mellitus but no history of chest pain, palpitation, arrhythmia, or familial history of SCD. She had history of hypothyroidism and normal thyroid function tests at present. Physical examination was unremarkable. The baseline ECG during sinus rhythm showed typical coved type J point and ST-segment elevation in right precordial leads and corrected QT interval of 396 ms (Figure 1A). A twelve-lead ECG obtained two days later revealed typical coved type ST-segment elevation in leads I and aVL (Figure 1B). An asymptomatic pause of 3.15 seconds was detected in 24-hour ambulatory ECG monitoring. The signal-averaged ECG showed the filtered QRS duration (fQRS) of 111 ms, duration of the low amplitude signal of less than 40 μV (LAS-40) of 41 ms, root-mean-square voltage of the signals in the last 40 ms (RMS-40) of 12 μV. Laboratory examinations, including cardiac marker, hematologic and biochemical tests, chest x-ray, transthoracic echocardiogram, and myocardial perfusion imaging and coronary angiography revealed no apparent cardiac diseases. DNA testing disclosed no SCN5A mutation.

After obtaining an informed consent, an electrophysiology study was performed in fasting state and sinus rhythm. The results of electrophysiology study showed AH interval of 102 ms and HV interval of 56 ms, and corrected sinus node recovery time (CSNRT) of 592 ms.

The programmed ventricular extrastimulation (S1: 600, 500, and 400 ms and using up to three extrastimuli from RV apex with no coupling interval of less than 200 ms) failed to induce any supraventricular or ventricular tachyarrhythmia.

In view of the frequent episodes of traumatic syncope and Brugada syndrome, a dual-chamber ICD (GEM III DR, Model 7275, Medtronic Inc., Minneapolis, MN, USA) with active atrial (CapSureFix Novus, Model 4076, Medtronic Inc., Minneapolis, MN, USA) and ventricular (SprintFidelis, Model 6949, Medtronic Inc., Minneapolis, MN, USA) leads were implanted. During 9-month follow-up, one episode of appropriate ICD therapy for ventricular fibrillation was detected. She also had two episodes of severe sinus bradycardia (HR<40 bpm) treated by backup ventricular pacing.

## Discussion

The typical ECG pattern in patients with Brugada syndrome includes ST-segment elevation in the right precordial leads associated with RBBB, normal QT duration, and mild conduction defects with prolonged PR and HV intervals. Recently, several cases of the Brugada syndrome with ST-segment elevation in inferior leads have been reported[2-7]. There is also one report of the patient with Brugada syndrome who had alternating ST-elevation in the right precordial leads and the high lateral leads [8].

Our patient represented another interesting case of combination of the typical coved type ST-elevation in the right precordial and the high lateral leads in the same patient. This patient had a highly aggressive Brugada syndrome (history of frequent traumatic syncope). The episodes of syncope in our patient can be explained by both tacharrhythmic and bradyarrhythmic events (sinus node dysfunction), as evidenced by appropriate ICD therapy for ventricular fibrillation and backup ventricular pacing for severe bradycardias during follow-up. Similarly high risk clinical features were reported for the patients with Brugada syndrome who had ST-segment elevation alternatively in the inferior and the right precordial leads [2-7].

Suggested cellular mechanisms that could account for the Brugada phenotype include a reduced density of the Na^+^ current and a denser transient outward current (I_to_) in the right ventricular epicardium than the left ventricular myocardium [9], thereby the transmural voltage gradient usually manifest itself in the right precordial leads. Alternating expression of the Brugada ECG phenotype in the high lateral and the right precordial leads may be explained by the biventricular involvement in this group of patients with Brugada syndrome. Other possibilities include differential vagal innervation in the right ventricular and lateral left ventricular walls.

Although our patient showed no SCN5A mutation, we could not check for other mutations, such as those reported in genes encoding for cardiac calcium channel and also in the glycerol-3-phosphate dehydrogenase 1-like gene [10,11]. This report suggests that the Brugada syndrome may have different electrocardiographic presentations within a single individual over a short period of time. The significance of these changes needs to be assessed in a prospective long term study.
